# Precise Control of Glioma Cell Apoptosis Induced by Micro-Plasma-Activated Water (μ-PAW)

**DOI:** 10.3390/mi13122145

**Published:** 2022-12-04

**Authors:** Yuhan Zhang, Xiaoxia Du, Qihao Shi, Wenxiang Xiao, Hua Li

**Affiliations:** School of Life and Environmental Sciences, Guilin University of Electronic Technology, Guilin 541004, China

**Keywords:** He micro-plasma jet array, tumor cell apoptosis, micromole level

## Abstract

To verify the existence of plasma with the potential to kill tumor cells, this paper designed a novel helium (He) micro-plasma jet array device and detected the concentration of typical long-lived reactive oxygen and nitrogen species (RONS) with oxidative activity generated by it. The paper described a new He micro-plasma jet array device consisting of nine flexible quartz capillaries with an inner diameter of 75 μm arranged in a 3 × 3 array. Sterilized ultrapure water (up water) was first treated with the He micro-plasma jet array device to activate it to form enriched RONS micro-plasma-activated water (μ-PAW), and then μ-PAW was added to the cell culture medium (with cells) to observe the proliferation of human glioma cells. The concentration of long-lived RONS, such as nitrate (NO_3_^−^), was detected according to Beer–Lambert’s law in combination with UV spectrophotometry as well as a color development method. The MTT Cell Proliferation and Cytotoxicity Assay Kit combined with the Hoechst Staining Kit were used to assess the proliferation status of the cells. The results showed that the range of RONS concentration variation could be controlled in the order of micromoles (µmol), while plasma-induced tumor cell death is apoptosis that does not affect the surrounding environment.

## 1. Introduction

Plasma is generated by electrical discharges at atmospheric pressure and is capable of triggering different physical phenomena as well as chemical reactions [1]. The essence of plasma production and its existence is the transformation of gas molecules into an approximately electrically neutral ionized gas consisting of a population of electrons and ions after they have acquired high energy under the effect of heating or strong electromagnetic fields. This highly ionized gas contains electrons, ions, atoms, molecules, reactive radicals, excited species, and rays [2] and therefore contains generally higher energy than other particles, such as ions and neutral particles. In cold atmospheric plasma (CAP), as only part of the gas is ionized, the temperature of heavy ions is relatively low because it is much lower than the temperature of electrons [3], which are close to room temperature and at least below 42 °C [2,4,5]. CAP was demonstrated to affect cells not only directly but also indirectly with the plasma pre-treatment solution [6]. In the past decade, CAP has become a separate field with high relevance to biomedical science. By precisely controlling the dose of reactive oxygen and nitrogen species (RONS) generated by precisely controlling the plasma activation time, CAP can act directly on human skin and tissues, resulting in a new plasma medicine technology with various applications, including skin disinfection [7,8,9], wound healing [10,11], sterilization [12,13], and cancer therapy [14,15].

As an intracranial malignant tumor accounting for nearly 50% of primary tumors, glioma is characterized by difficulty in eradication, a high recurrence rate, and poor prognosis [16,17,18]. The current treatment for glioma is based on radical surgery for malignant tumors, combined with a post-operative radiotherapy and chemotherapy plan for glioma. There are also scholars, such as Rühm et al., who used molecular stereotactic biopsy to accurately locate gliomas [19]. However, the current comprehensive treatment has certain side effects, such as incomplete removal by surgery, destruction of functional areas, and indiscriminate killing of normal nerve cells and glioma cells by radioactive elements during radiation therapy [20,21]. Chemotherapy, in contrast, is not only limited by the drug tolerance of glioma (e.g., platinum-based chemotherapeutic agents, a class of chemotherapeutic agents that affect the cell cycle, are not effective, because gliomas are resistant to platinum-based drugs [22]) but also decreases neurocognitive and other functions [23,24].

The gaseous active species generated by plasma can undergo a series of chemical reactions with aqueous solutions or cell culture medium to produce liquid active species, including NO_3_^−^, NO_2_^−^, H_2_O_2_, OH, and others, which has been known as plasma-activated water (PAW) with redox activity [25,26]. The active species in PAW can enter tumor cells and affect their developmental functions and metabolic mechanisms [27]. Plasma has become a focus of attention because it has been found to be effective and stable in destroying tumor cells, bringing a new light to the treatment and prognosis of cancer [28]. Considering the distinctive growth site of glioma, direct plasma treatment is impractical because of the high-voltage discharge to surrounding tissues and gas transport [29]. Therefore, in this paper, a novel helium (He) micro-plasma jet array device was designed to treat human glioma cells in the form of micro-plasma-activated water (μ-PAW). By applying AC high voltage to a 0.2-mm-inner-diameter hollow metal capillary tube to generate plasma, RONS with oxidation activity were generated in the plasma, and instead of directly contacting the surface of the treated object, the voltage first passes through the array of nine flexible quartz capillaries with an inner diameter of 75 μm connected at the lower end and finally enters the treated object (sterilized ultrapure water (up water) used as the medium in this paper). The device can reduce the discharge voltage on the one hand as well as play the role of precise control of the RONS dose on the other hand [30]. To elucidate the mechanisms leading to cell death and the role of RONS in the culture medium, this paper shows the correlation between the concentration of plasma-generated RONS and the inactivation of human glioma cells by measuring the concentration of long-lived active species, such as nitrate (NO_3_^−^), hydrogen peroxide (H_2_O_2_), and nitrite (NO_2_^−^), in μ-PAW.

## 2. Materials and Methods

### 2.1. Cell Line and Culture

Human glioma cells (H4, CCTCC-GDC0128, China Center for Type Culture Collection) were cultured under standard cell culture conditions at 37 °C with 5% CO_2_ in complete medium consisting of 89% (*v*/*v*) high-glucose-type Dulbecco’s Modified Eagle Medium (DMEM, Cytiva HyClone Laboratories, Pasching, Austria.), 10% (*v*/*v*) fetal bovine serum (FBS, Beijing Labgic Technology Co., Ltd. Beijing, China.), and 1% (*v*/*v*) antibody solution (penicillin–streptomycin solution, Cytiva HyClone Laboratories, Pasching, Austria).

### 2.2. Experimental Setup

**Figure 1:**
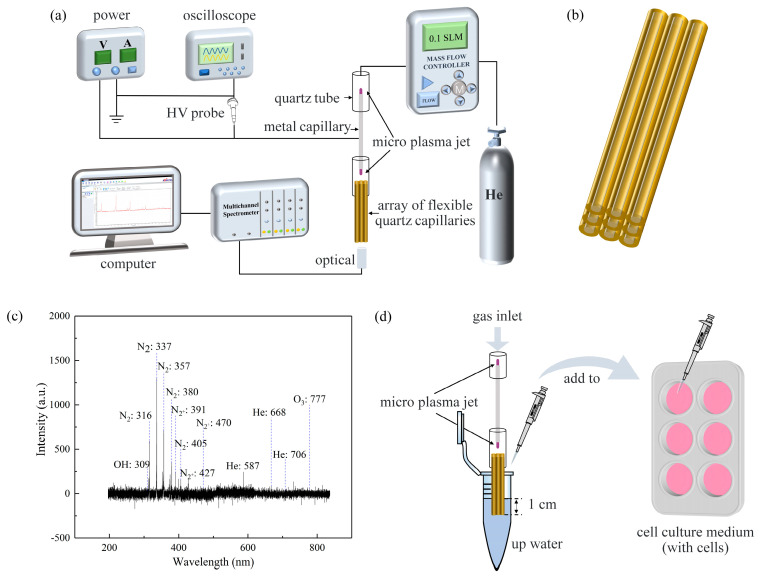
(**a**) Model diagram of the He micro-plasma jet array device. (**b**) Side view of the array of flexible quartz capillaries. Flexible quartz capillaries with an inner diameter of 75 µm and an outer diameter of 365 µm were arranged in a 3 × 3 array. (**c**) Emission spectra of the He micro-plasma jet array device measured at the nozzle of a flexible quartz capillary. (**d**) Schematic diagram of μ-PAW indirect treatment: 1 mL of sterilized up water activated using the He micro-plasma jet array device for 1, 5, and 10 min to obtain μ-PAW enriched with varying concentrations of RONS was added to human glioma cells in DMEM immediately and incubated in 6-well or 96-well plates for 24 or 48 h. μ-PAW: micro-plasma-activated water; RONS: reactive oxygen and nitrogen species; up water: ultrapure water; DMEM: Dulbecco’s Modified Eagle Medium.

**Figure 2:**
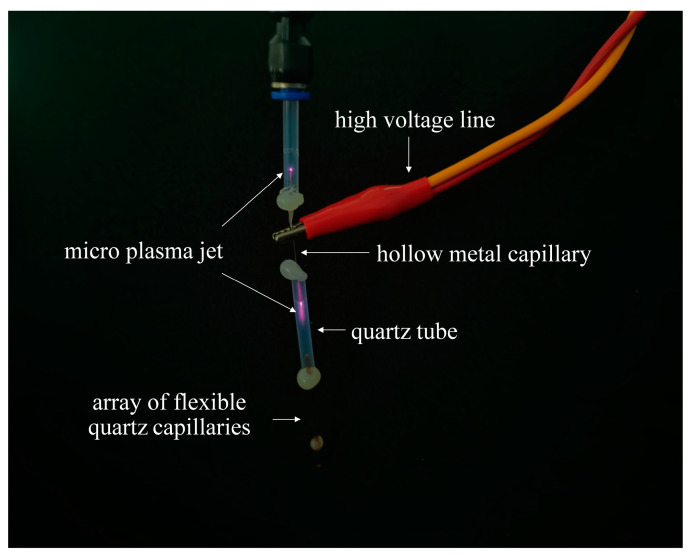
Diagram of He micro-plasma jet. The micro-plasma jet with a gas flow rate of 0.1 slm, an AC voltage of 5 kV, and a frequency of 10 kHz. He: helium; slm: standard liters per minute.

As shown in Figure 1a, the device was based on single-electrode discharge, using a hollow metal capillary tube with an inner diameter of 0.2 mm as the single electrode. An AC voltage was applied to the single electrode to ionize He to generate a plasma jet. Experimental studies have been conducted and have found that He gas is used as a carrier gas due to its low voltage to induce plasma [31] and it has more active material than other candidate gases (such as argon) [32]. The upper end of the hollow metal capillary tube was connected to a He gas tube through a quartz tube, and the lower end was connected to flexible quartz capillaries through a quartz tube. As shown in Figure 2, the plasma jet was generated only at the two ends of the hollow metal capillary tube, and outside the 3 × 3 array of flexible quartz capillaries the plasma jets were not visible, which not only acts as a discharge device but also as part of the gas path: the ionized RONS are blown out onto the surface of the treated object by the He gas stream (the sterilized up water used as the treated object). The side view of the flexible quartz capillaries with an inner diameter of 75 μm is shown in Figure 1b. The device used 9 flexible quartz capillaries with an inner diameter of 75 μm, an outer diameter of 365 μm, and a length of 40 mm arranged in a 3 × 3 array, and RONS generated under the action of He flow reach the surface of the treated object (up water used as the treated object). A spectrometer, within a wavelength range of 200–825 nm, was used to determine the active species produced by the He micro-plasma jet array device. Detection is performed by a fiber optic probe fixed in a bracket, facing the discharge gap and maintained in a horizontal position, plasma radiation light intensity by the converging lens on the fibre optic probe convergence after the light signal, by the fiber optic transmission signal, through the steps of separation and conversion, and the data is finally collected and display using a computer [33]. Figure 1c shows the optical emission spectrum (OES) of the He micro-plasma jet array device with a gas flow rate of 0.1 standard liters per minute (slm), an AC voltage of 5 kV, and a frequency of 10 kHz. The spectrometer and fiber optic probe were purchased from Avantes Technology Ltd. (Beijing, China). The fiber optic probe was placed at a distance of 1 mm from the end of the He micro-plasma jet array nozzle. Data were acquired at an integration time of 500 ms.

### 2.3. PAM Treatment

In this paper, cells were first cultured overnight in 6-well or 96-well plates, grown adherent to the wells. Figure 1d shows a schematic diagram of this experiment with an AC voltage of 5 kV and a frequency of 10 kHz. The flow rate of He was set to 0.1 slm. After human glioma cells were attached, 1 mL of sterilized up water was activated by the device, as shown in Figure 1a, for 1, 5, and 10 min to obtain μ-PAW enriched with varying concentrations of RONS. Subsequently, human glioma cells with μ-PAW were incubated in 6-well or 96-well plates for 24 or 48 h. The cell growth states were obtained using fluorescence microscopy.

### 2.4. Determination of RONS, Temperature, and pH

In this paper, NO_3_^−^, H_2_O_2_, and NO_2_^−^ were used to study the concentration of long-lived RONS in μ-PAW using a UV spectrophotometer, which is important to deepen the understanding of the mechanism of cell death caused by plasma. The reaction of sodium hydroxide (NaOH) and nitric acid (HNO_3_) can produce stable NO_3_^−^, whose maximum absorption wavelength is 202.5 nm [34]. In an acidic medium, H_2_O_2_ can instantly associate with molybdate to form a peroxomolybdic acid complex that has an absorption peak at 330 nm. To avoid the spectral interference from excess molybdate ions, 352.5 nm was used for spectroscopic quantification [35,36]. Nitrite was determined with the colorimetric method using *N*-1-naphthalene ethylenediamine hydrochloride: under the condition of hydrochloric acid acidification, nitrite reacts with p-aminobenzene sulfonic acid and then combines with *N*-1-naphthalene ethylenediamine hydrochloride (produced by the action of naphthalene ethylenediamine and hydrochloric acid) to form a purple-red dye with a maximum absorption value of 550 nm [37,38,39]. The temperature of the micro-plasma jet at different times was measured using an infrared thermometer (DECTMM520C, DELIXI ELECTRIC LTD). We use a pH meter (LE438, FiveEasy Plus, Mettler Toledo, Columbus, OH, USA) to measure the pH of μ-PAW at different activation times and of the liquid mixed by adding it to the 50% (*v*/*v*) medium (containing the cells).

### 2.5. Cellular Viability of μ-PAW-Treated Cells In Vitro

MTT, also known as thiazole blue, is a yellow granular dye that can enter the cell through the cell membrane. It can be metabolized by enzymes, such as succinate dehydrogenase, in the mitochondria of living cells to reduce the tetrazolium ring in the MTT molecule and break the ring to form a crystalline dark-purple formazan, the amount of which is proportional to the cell viability [40]. To determine the inhibitory effect of the He micro-plasma jet on cell proliferation, the MTT Cell Proliferation and Cytotoxicity Assay Kit (Beyotime, Jiangsu, China) was used for the analysis of cellular viability. MTT is insoluble in water but can be completely dissolved in the kit’s own dissolved liquid of formazan. The more and faster the cells proliferate, the higher the absorbance; the greater the cytotoxicity, the lower the absorbance. Resuspended human glioma cells were inoculated in 96-well plates containing 100 µL of cell culture medium overnight to maintain proper well attachment and stable growth. Next, 10 µL out of 1 mL of μ-PAW activated for 1, 5, and 10 min was added to the 96-well plates containing 100 µL of cell culture medium and incubated for 24 or 48 h. Further, 10 μL of 5 mg/mL MTT solution was added to each well of the plates and incubated for 4 h. Subsequently, 100 μL of formazan was added to each well for about 3–4 h until it was completely dissolved. The absorbance of the solution was measured at 570 nm using a microplate photometer (Multiskan FC, Thermo Fisher Scientific, Waltham, MA, USA). The cell survival rate was calculated as in Equation (1):

Cell survival rate = [(As − Ab)/(Ac − Ab)] × 100%(1)

where As is the absorbance of experimental wells, Ac is the absorbance of control wells (PAW not included), and Ab is the absorbance of blank group wells (PAW and cells not included).

### 2.6. Cellular Apoptosis of μ-PAW-Treated Cells In Vitro

Hoechst stain can pass through the cell membrane of cells, and the main mechanism of action is to bind to DNA in the nucleus so that cells in different states show different staining conditions. When apoptosis occurs, the chromatin solidifies. The staining of non-apoptotic cells shows uneven distribution of fluorescence and different shades of brightness, so Hoechst staining has significant advantages in distinguishing apoptotic cells, non-apoptotic dead cells, and normal living cells. A plasma jet can inhibit cell proliferation [41], but whether the proliferation inhibition is caused by the induction of cell regulation or necrosis can be analyzed by using the Hoechst Staining Kit (Beyotime, Jiangsu, China). After Hoechst 33,258 staining, the nuclei of normal cells are blue when viewed under a fluorescent microscope, while the nuclei of apoptotic cells are dense and densely stained or fragmented and densely stained, with a somewhat whitish color. Resuspended human glioma cells were inoculated in 6-well plates containing 1 mL of cell culture medium overnight to maintain proper well attachment and stable growth. Next, 1 mL of μ-PAW activated for 1, 5, and 10 min was added to the 6-well plates containing 1 mL of cell culture medium and incubated for 24 or 48 h. Staining was performed according to the steps provided in the product instructions and finally detected at E_x_ = 350 nm and E_m_ = 460 nm under an inverted fluorescence microscope (Ts2FL, Nikon, Tokyo, Japan).

## 3. Results

### 3.1. Measurement of the Optical Emission Spectrum (OES)

The spectral lines and intensity of the emission spectrum were tested directly by the manufacturer. The identification of the spectral lines of the emission spectrum was performed according to [40]. The presence of a large number of gaseous active species in the He micro-plasma jet array was seen, as shown in Figure 1c. The micro-plasma jet generated during the ionization of He discharges was analyzed and measured, and spectral lines were analyzed mainly in three categories: the first one is the hydroxyl spectral line (OH: 309 nm); the second one is the first negative band system of nitrogen B^2^Σu^+^-X^2^Σu^+^, including nitrogen ions (N_2_^+^ (0, 0): 391 nm), (N_2_^+^ (0, 3): 405 nm), (N_2_^+^ (0, 1): 427. 5 nm), and (N_2_^+^ (0, 2): 470.82 nm); and the third one is (3^3^D-2^3^P) 587 nm, (He 3^1^D-2^1^P) 668 nm, and (He 3^3^S-2^3^P) 706 nm for the He spectral line. The gaseous active species generated by plasma can undergo a series of chemical reactions with aqueous solutions or cell culture media to produce liquid active species, including NO_3_^−^, NO_2_^−^, H_2_O_2_, and OH, which can react with cells and induce programmed cell death [25,26]. Therefore, in a later section, we detected the long-lived liquid active species in μ-PAW.

### 3.2. Measurement of RONS, Temperature, and pH

**Figure 3:**
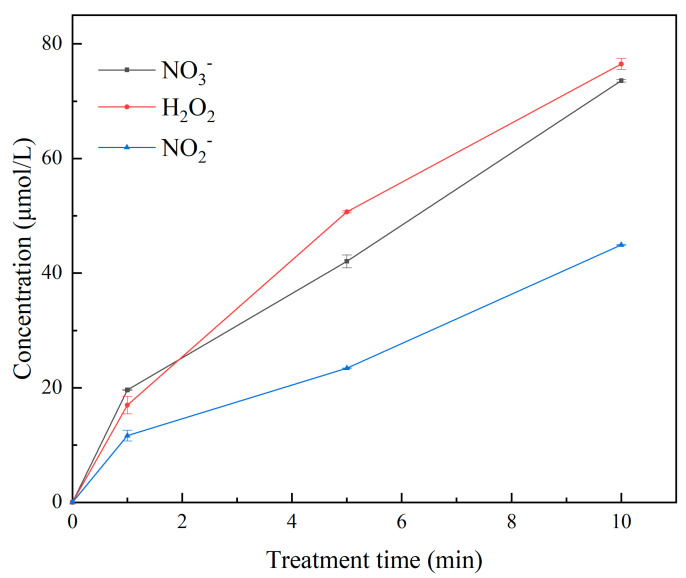
Concentrations of long-lived RONS, such as NO_3_^−^, H_2_O_2_, and NO_2_^−^ in μ-PAW at 1, 5, and 10 min of activation were measured according to Beer–Lambert’s law in combination with UV spectrophotometry as well as a color development method.

**Figure 4:**
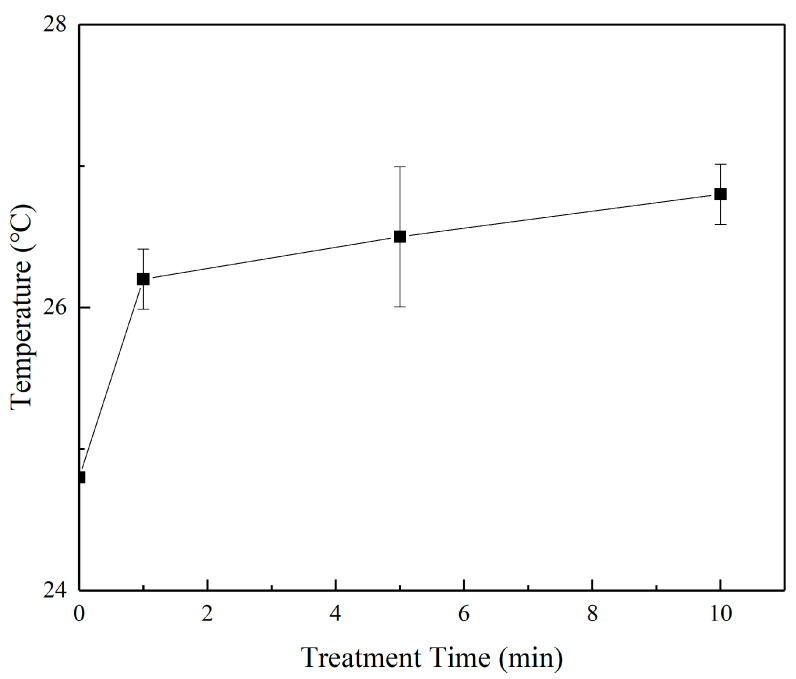
Measurement of the jet temperature of the helium micro-plasma jet array device using an infrared thermometer under different times of activation.

**Figure 5:**
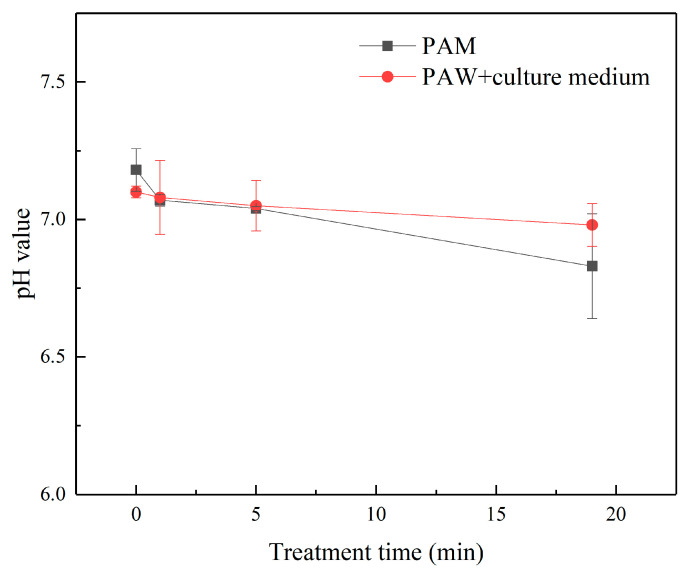
Measurement of the pH of μ-PAW and its mixed solution with 50% (*v*/*v*) cell culture medium (containing cells) using a pH meter under different times of activation.

The findings of Zhang and other groups show that a micro-plasma jet can trigger oxidative stress in cells [42,43,44,45,46]. The electron concentration and the reduced electric field gradually increase with the extension of the μ-PAW activation time [47,48]. The concentration of RONS corresponding to μ-PAW at different treatment times is shown in Figure 3. It can be clearly seen that the concentration gradually increased with increasing μ-PAW activation time, showing an approximately linear relationship [49]. The concentration of NO_3_^−^, H_2_O_2_, and NO_2_^−^ in µ-PAW activated for 1 min was 19.636 µmol/L, 16.991 µmol/L, and 11.673 µmol/L, respectively. With the extension of the activation time of μ-PAW, the concentration of NO_3_^−^, H_2_O_2_, and NO_2_^−^ increased to 42.061 µmol/L, 50.708 µmol/L, and 23.438 µmol/L at 5 min, respectively. When the activation time reached 10 min, the concentration of NO_3_^−^, H_2_O_2_, and NO_2_^−^ increased to 73.576 µmol/L, 76.484 µmol/L, and 44.939 µmol/L, respectively. It can be clearly seen that the increase in RONS of different species can be maintained at the micromole (µmol) level, achieving precise control in terms of dose. We measured the temperature of the plasma jet using an infrared thermometer, as shown in Figure 4, and the experimental results showed that the temperature of the micro-plasma jet reached 26.8 °C after 10 min, which was below 42 °C. The pH of PAW has a wide range of variation (because PAW contains active substances, such as H_2_O_2_, the pH of different concentrations of H_2_O_2_ ranges from 4.5 to 6.2 [43]), the plasma solution in a previous study by Fridman et al. ranged from 5.0 to 6.0 [50], as shown in Figure 5, but the range of variation can be maintained at 0.1 when the μ-PAW generated using the device we designed is mixed with the cell culture medium containing cells.

### 3.3. Detection of Cell Proliferation

**Figure 6:**
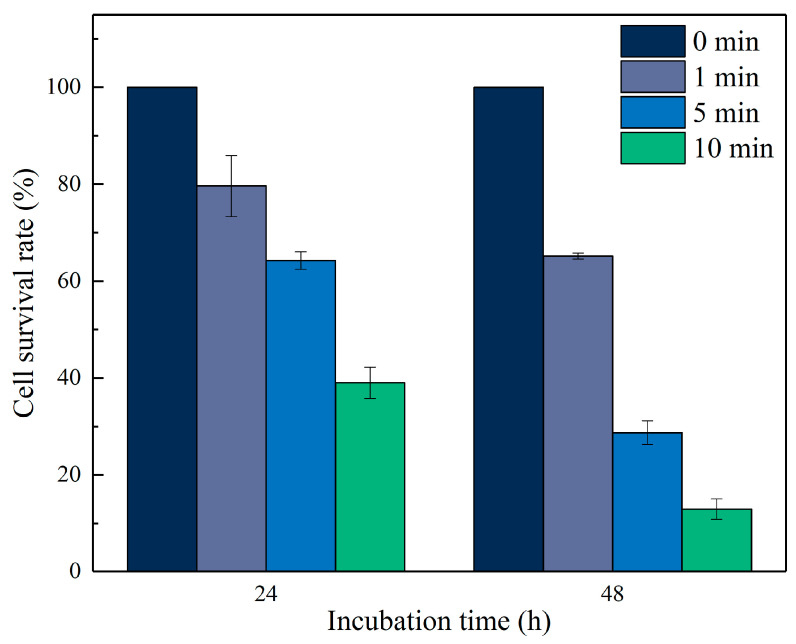
Cell survival rate of human glioma cells incubated in 96-well plates for 24 or 48 h with equal amounts of μ-PAW activated by the He micro-plasma jet device for 1, 5, and 10 min. “0 min” represents sterilized up water not activated by the He micro-plasma jet array device.

Human glioma cells were treated with μ-PAW after different times of activation after 24 or 48 h of incubation, and their cell survival rate was measured. As shown in Figure 6, the proliferation inhibition of human glioma cells by μ-PAW exhibited a clear time-dependent (the time of activation of μ-PAW) and dose-dependent manner (the dose of RONS in μ-PAW). The cell survival rate after 24 h of incubation reduced to 79.635%, 64.257%, and 39.007% after the addition of μ-PAW activated for 1, 5, and 10 min to the medium, respectively. When μ-PAW activated for 1 min was added to the cell medium and the cells were continued to be cultured for 24 h, the growth of the cells was already more significantly inhibited. The cell survival rate after 48 h incubation reduced to 65.156%, 28.715%, and 12.940% corresponding to the addition of μ-PAW activated for 1, 5, and 10 min to the medium, respectively. In Figure 6, “0 min” means that μ-PAW activated for 0 min was added to the medium; in other words, sterilized up water was added to the medium as a control.

### 3.4. Detection of Cell Apoptosis

**Figure 7:**
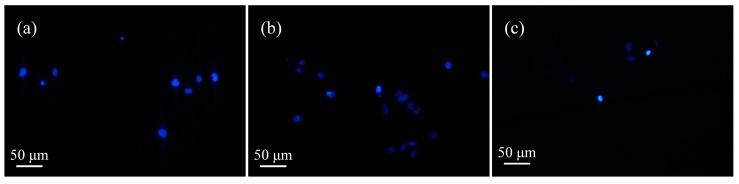
Apoptosis of human glioma cells after 24 h incubation in 6-well plates with equal amounts of μ-PAW activated by the He micro-plasma jet device for (**a**) 1 min, (**b**) 5 min, and (**c**) 10 min.

**Figure 8:**
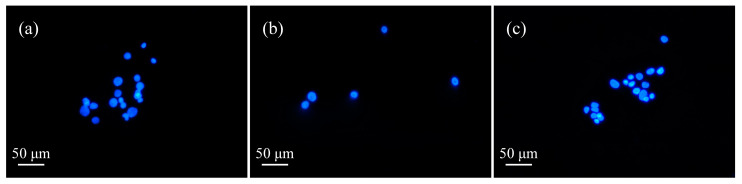
Apoptosis of human glioma cells after 48 h incubation in 6-well plates with equal amounts of μ-PAW activated by the He micro-plasma jet device for (**a**) 1 min, (**b**) 5 min, and (**c**) 10 min.

As can be seen from the figures, in the control group, after the addition of unactivated μ-PAW (Figure 7a and Figure 8a), the cells mostly appeared light blue, but with the extension of the activation time of μ-PAW, the appearance of bright-blue cells could be clearly observed, indicating that with the addition of μ-PAW, the cells underwent apoptosis after incubation for a specific time. It can be concluded that the cell death after He micro-plasma jet treatment is apoptosis that does not affect the surrounding environment.

## 4. Conclusions

In summary, plasma-generated gaseous RONS can undergo a series of chemical reactions with aqueous solutions or cell culture media to produce liquid RONS, including NO_3_^−^, NO_2_^−^, H_2_O_2_, OH, and others, which are known as PAW with redox activity. As the activation time of μ-PAW increased, the RONS concentration also gradually increased and showed an approximately linear relationship. Based on this, in this paper, a He micro-plasma jet array device was proposed and its antitumor effect was demonstrated on human glioma cells in vitro. Compared to conventional plasma jet devices, the He micro-plasma jet array device is small and, at the same time, produces a small dose of active species. It is for this reason that the dose of RONS produced can be better controlled, while keeping the basic experimental conditions constant, and it was verified that it can be controlled at the micromole level. Therefore, it can reduce the impact on the surrounding tissues compared to conventional oncology treatment protocols. The cell survival rate can be reduced by more than 80% after μ-PAW activated by the micro-plasma jet array device for 10 min is added to the cell culture medium containing cells and incubated for 48 h. It can be assumed that the He micro-plasma jet array device can be induced to reduce the cell survival rate of human glioma cells in a time-dependent and dose-dependent manner. Even when the He micro-plasma jet array device is not in direct contact with human glioma cells but in the form of μ-PAW, its antitumor effect can be maintained, which may be related to the oxidative activity of the active species generated by He micro-plasma jet array device and its penetration effect. This work offers a potential application for the effective and precise control of the dose of RONS generated by the He micro-plasma jet array device for the treatment of cancer in vitro.
